# Supplementary material for: Knowledge gaps and structural barriers to prescribing pre-exposure prophylaxis among healthcare providers in North Louisiana: A cross-sectional study
Source: PLoS One. 2026 May 26;21(5):e0346890. doi: 10.1371/journal.pone.0346890 (PMC13210140; doi:10.1371/journal.pone.0346890)
Supplement: Attachment — Submitted filename: Reviewer matrix.docx [file pone.0346890.s002.docx]

**REVIEWER’S COMMENTS**

PLOS ONE Global Public Health.

Knowledge Gaps and Structural Barriers to Prescribing Pre-Exposure Prophylaxis Among Healthcare Providers in North Louisiana: A Cross-Sectional Study.

| **S/N** | **Section** | **Comments from the reviewer** | **PI’s response** | **Line number** |
| --- | --- | --- | --- | --- |
| 1. | **Title** | OK | NA | NA |
| 2. | **Abstract** | Write the section of abstract into strcutured paragraphs |  | 23-39 |
|  |  | Include p-values of the variables which showed significance |  | 34-35 |
| 3. | **Introduction** | State in number rates of new HIV diagnoses in the country |  | 42 |
|  |  | State situation globally, regional and locally as far as Pre- exposure prophylaxis is concern |  | 42-63 |
| 4. | **Methodology** | State the high HIV burden in the region(North Louisiana) |  | 68 |
|  |  | What is the ideal sampling technique you have used in the selection of study participants |  | 71-75 |
|  |  | Why did you remained with only 102 out 104? |  | 74-75 |
|  | **Measures** | Where did you adapt this set of questions  How did you measure knowledge and practice without using scale? |  | 79-82 |
|  | **Inclusion criteria** | What were the inclusion criteria |  |  |
|  | **Exclusion criteria** | What were the exclusion criteria |  |  |
| 5. | **Data management and analysis** | Show what was the collection tools used in this study?? It is not clear stating electronically |  | 108 |
|  |  | What made you choosing Logistic regression as the model for analysis??? |  | 112 |
| 6. | **Ethical approval** | Show how the confidentiality and privacy was done as well as how participants were enrolled ethically |  | 117-119 |
| 7. | **Results** | Include the p-values for the significantly associated variables |  | 137-138 |
|  |  | Provide N within each percentage composition in the results |  | 121-132 |
| 8. | **Discussion** | Please compare findings with other studies around the world |  | 156-158 |
|  |  | Please provide countries and year of study in which specific studies were conducted |  | 156-240 |
|  |  | Provide long form of OB/GYN |  | 215 |
| 9. | **Conclusion** | Provide recommendations in separate section. |  | 244-246 |
| 10. | **Acknowledgements** | OK | NA | NA |
| 11. | **General Comments** | Do you think 102 participants are essentially enough for publication and later be generalizable to the country. |  |  |
|  |  | Why was study not a mixed design??? |  |  |
|  |  | What were the limitations of the study and how to mitigate them |  |  |
|  |  | Provide each authors contribution as well |  |  |
