# Supplementary material for: Knowledge gaps and structural barriers to prescribing pre-exposure prophylaxis among healthcare providers in North Louisiana: A cross-sectional study
Source: PLoS One. 2026 May 26;21(5):e0346890. doi: 10.1371/journal.pone.0346890 (PMC13210140; doi:10.1371/journal.pone.0346890)
Supplement: Attachment — Submitted filename: Response to Reviewers.docx [file pone.0346890.s005.docx]

**Reviewer question:** **I would like you to include under the "Participant and Recruitment" section, the sample size calculated or estimated for the study, including your assumptions.**

Response: Thank you for your question and comment. We agree that reporting how the sample size was estimated would improve the transparency and rigor of our methodology. We have added the following paragraph and a new reference used to estimate the study sample size required for our study, and we have included details in the “Participants and Recruitment” section.

*“Although convenience sampling was employed within a single academic medical center, an estimated sample size was considered to ensure adequate precision of proportion estimates. Since there were no prior estimates of the proportion of providers reporting the outcomes of interest (e.g., PrEP prescribing), a conservative assumed proportion of 50% was used, as recommended by Lwanga and Lemeshow [7], in standard sample size formulas for estimating proportions in cross-sectional studies, because this produces the largest required sample size. Assuming a 95% confidence level and a margin of error of ±10 percentage points, the minimum estimated sample size required was approximately 96 participants. A total of 104 healthcare providers completed the survey. Incomplete responses were excluded using listwise deletion, resulting in a final analytic sample of 102.”*
